# Species specificity preliminary evaluation of an IL‐4‐based test for the differential diagnosis of human echinococcosis

**DOI:** 10.1111/pim.12695

**Published:** 2020-02-07

**Authors:** Linda Petrone, Werner C. Albrich, Francesca Tamarozzi, Manuel Frischknecht, Maria Angeles Gomez‐Morales, Antonella Teggi, Matthias Hoffmann, Delia Goletti

**Affiliations:** ^1^ Translational Research Unit Department of Epidemiology and Preclinical Research National Institute for Infectious Diseases Lazzaro Spallanzani–IRCCS Rome Italy; ^2^ Division of Infectious Diseases and Hospital Epidemiology Cantonal Hospital St. Gallen St. Gallen Switzerland; ^3^ Foodborne and Neglected Parasitoses Unit Department of Infectious Diseases Istituto Superiore di Sanità Rome Italy; ^4^ Department of Infectious and Tropical Diseases Sant'Andrea Hospital University of Rome "Sapienza" Rome Italy; ^5^ Medical Department Infectious Diseases Services Kantonsspital Olten Olten Switzerland

**Keywords:** cytokine, *Echinococcus* spp, ELISA, Enzyme‐linked immunosorbent assay, hydatidosis, immunodiagnosis, serodiagnosis

## Abstract

The diagnosis of cystic echinococcosis (CE) is based on imaging, while serology is a complementary test of particular use when imaging is inconclusive. Serology has several limitations. Among them, false‐positive results are often obtained in subjects with alveolar echinococcosis (AE), rendering difficult the differential diagnosis. We set up an immune assay based on IL‐4‐specific production after stimulating whole blood with an antigen B (AgB)‐enriched fraction from *E granulosus* that associates with CE and CE cysts in active stage. We aimed to evaluate potential cross‐reactivity of this test using samples from patients with AE. Twelve patients with AE were recruited; IL‐4 levels ranged from 0 to 0.07 pg/mL. Based on the previously identified cut‐off of 0.39 pg/mL using samples from patients with CE, none of samples from AE patients scored positive. In contrast, almost 80% of samples from AE patients scored positive in serology tests based on different *E granulosus*‐derived antigenic preparations. Our preliminary data show that this experimental whole‐blood assay has no cross‐reactivity in our cohort of patients with AE, in turn indicating a high specificity of the assay for CE diagnosis. This result supports further work towards the development of improved diagnostic tests for CE.

## 1 INTRODUCTION

Cystic echinococcosis (CE), caused by *Echinococcus granulosus* sensu lato, is a chronic and complex zoonosis, characterized by the growth of parasitic cysts in different organs. Diagnosis of CE is based on imaging techniques, mainly ultrasound (US) for abdominal locations.^1^ Serology supports imaging in doubtful cases but cannot be used alone for CE diagnosis in the absence of a compatible lesion identified by imaging.^1^, ^2^ Moreover, serology results are influenced by several factors, including cysts characteristics and occurrence of current or previous treatment.^3^ Therefore, the correct interpretation of serology results is challenging. The differential diagnosis of CE cysts on imaging may be broad, ranging from harmless biliary cysts to neoplasms, and includes hepatic lesions caused by *Echinococcus multilocularis*, causing alveolar echinococcosis (AE).^4^, ^5^ Differentiation between these two infections is pivotal, as AE and CE greatly differ in terms of disease progression, management and prognosis.^4^, ^5^, ^6^ The geographical distribution of CE and AE greatly overlaps in central Asia and China, making the differential diagnosis of these two conditions particularly challenging. This is further complicated by the high rate of cross‐reactivity of serological tests (50%‐100%).^7^, ^8^, ^9^

Several approaches, such as the use of different sources of antigens and different readout systems, have been attempted to overcome the serology shortfalls.^9^, ^10^, ^11^, ^12^, ^13^, ^14^ The most commonly used serological tests for human CE are based on the detection of IgG antibodies against *E granulosus* hydatid cyst fluid (HCF). These tests show an 80%‐99% sensitivity with variable specificity (60%‐97%), while tests detecting IgG against purified or recombinant antigens show a better specificity (80%‐100%) but lower sensitivity (38%‐93%).^9^, ^15^ The discrimination between CE and AE on serology may not be clear in up to about 25% of cases even using specific tests, such as band‐pattern evaluation of HCF‐based immunoblotting and *E multilocularis*‐specific Em2plus‐ or Em18‐based serological assays,^14^, ^16^ making the results of these tests not always reliable to distinguish these two aetiologies in clinical practice.

Cytokines have been considered the basis of potential tools for the diagnosis and clinical management of CE, and associations between cytokine responses and some clinical features have been reported.^17^, ^18^, ^19^, ^20^, ^21^, ^22^ In this context, we recently set up an immune‐based test measuring the IL‐4 production after stimulating whole blood with an enriched fraction of antigen B (AgB—the most abundant antigen of HCF) or AgB peptides.^11^, ^12^ In this whole‐blood test, higher levels of IL‐4 associated with CE infection (71.4% sensitivity, 93.3% specificity) and with the presence of active cysts (84.6% sensitivity, 92% specificity).^12^ However, cross‐reactivity with AE has not been tested. Therefore, the aim of this study was to evaluate the specificity of the experimental IL‐4 test based on AgB‐enriched fraction for CE diagnosis, analysing samples from patients with AE from Eastern Switzerland, where AE is endemic.^23^

## 2 MATERIALS AND METHODS

Approval was granted from the Ethics Committees of the National Institute for Infectious Diseases (INMI), Rome, Italy (parere 59/2014), and of the canton of St. Gallen, Switzerland (EKSG 14/121). AE patients, either with a new or old diagnosis, who presented at a visit in the project timeframe and signed the informed consent, were prospectively and consecutively enrolled at the Kantonsspital St.Gallen between May 2015 and October 2017. AE was diagnosed based on most likely place of exposure, clinical presentation, serology and imaging. The case definition of AE followed the diagnostic criteria described by the WHO‐Informal Working Group on Echinococcosis.^6^ Based on these criteria, the AE patients enrolled were all defined as probable AE cases having clinical and epidemiological history, typical imaging findings and serology positive for AE with two tests. HIV‐positive subjects or taking immunosuppressive drugs were excluded.

Blood samples and clinical information were collected from each patient. Serology was carried out at the time of performing the experimental whole‐blood test (or within 6 months) as part of clinical routine at the Institute of Parasitology of the University of Zürich. Serological tests were performed according to manufacturer instructions or as previously reported,^24^ and were as follows: HCF‐enzyme immunotest (EIA), EgP‐EIA, Em2plus‐EIA, Em18‐EIA; AgB‐electro‐immuno‐transfer blot. All serological tests were in‐house assays^24^ with the exception of the commercial Em2plus‐EIA (Bordier Affinity Products). Unfortunately, the leftover serum was not available for further analyses.

**Figure 1:**
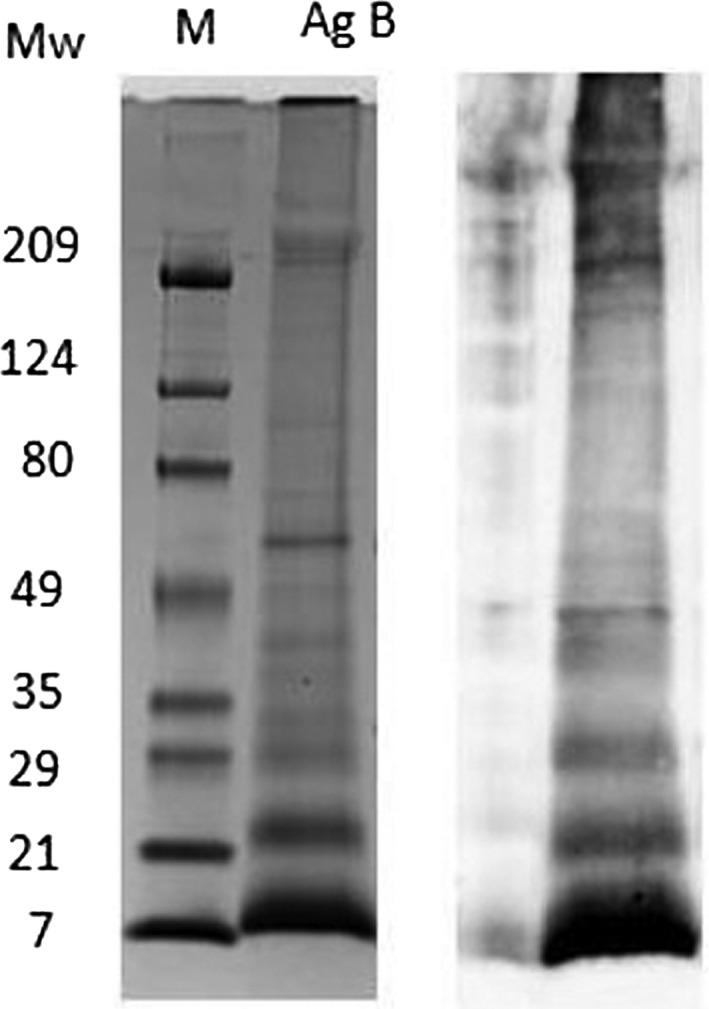
Analysis of AgB preparation. AgB‐enriched fraction was subjected to 4%‐20% SDS‐PAGE under reducing conditions and stained with Coomassie blue (left part) or transferred to nitrocellulose paper and incubated with a serum from a person with cystic echinococcosis (right part). AgB: antigen B‐enriched fraction; Mw M, prestained standard of molecular weight markers

For the whole‐blood test, AgB‐enriched fraction was purified from HCF obtained aseptically from echinococcal cysts of sheep from Sardinia (Italy). A purified AgB‐enriched fraction preparation was obtained after boiling HCF for 15 min; the sample was then centrifuged at 50 000 *g* at 4°C for 1 h. Protein content was determined by protein assay (Bio‐Rad). Protein integrity and analysis of AgB major bands have been performed through a 4%‐20% gradient gel (Figure 1, left part) followed by Western blot using as primary antibody the serum from a patient with CE (Figure 1, right part). In the gel, as well as in the Western blot analysis, bands corresponding to AgB are evident as they appear as molecular weight multiples of 8kDa (i.e. 8, 16, 24, 32 kDa) (Figure 1C). Whole blood was stimulated or not (negative control) with AgB‐enriched fraction and staphylococcal enterotoxin B (SEB, positive control); supernatants were sent to INMI for batch‐wise IL‐4 determination by ELISA, as previously described.^12^ Laboratory personnel was blinded to the patient diagnosis.

The whole‐blood test cut‐off for positivity of ≥ 0.39 pg/mL (IL‐4 concentration upon stimulation with AgB‐enriched fraction minus IL‐4 concentration in negative control) was previously established through receiver operator characteristic curve analysis of data from healthy donors and well‐characterized patients with CE.^12^ An indeterminate result was defined as an IL‐4 level < 4pg/mL in response to the SEB independently of the response to AgB of the same sample. IL‐4 results were expressed as medians and interquartile ranges. The AgB‐enriched fraction used in Petrone et al 12 was purified as described above, and antigen yield was 570 µg/mL. The gel images of the AgB‐enriched fraction batches were compared and showed that the AgB‐enriched fraction is a well‐purified preparation (data not shown).

## 3 RESULTS

**Figure 2:**
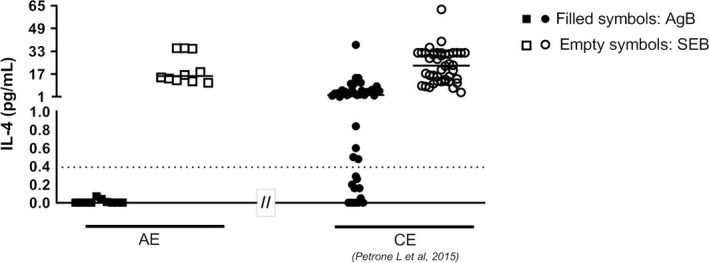
Analysis of whole‐blood IL‐4 test. Patient with AE have negative results on the whole‐blood IL‐4 test. AE patients (squares) showed low or no IL‐4 levels in response to AgB‐enriched fraction (filled squares) compared with CE patients (filled circles) previously evaluated in Petrone et al^12^ AE patients (squares) showed similar IL‐4 levels in response to the SEB‐positive control (empty squares) compared with CE patients (empty circles) previously evaluated in Petrone et al^12^ Horizontal bars represent medians. IL‐4 concentrations were determined by ELISA. Cut‐off = 0.39 pg/mL determined in Petrone et al^12^. AE: alveolar echinococcosis; CE: cystic echinococcosis; AgB: antigen B; SEB: staphylococcus enterotoxin B: IL: interleukin

We enrolled 12 patients with AE. Their demographic and clinical characteristics at the time of performing the whole‐blood test are shown in Tables 1 and 2. All but one patient with AE had received medical therapy with albendazole (median: 2 years, range 10 days to 8 years), and 10/11 were still receiving albendazole at the time of performing the whole‐blood test, whereas in one patient the treatment was discontinued 3 years before performing the whole‐blood test; 4 had undergone nonradical surgical resection of liver lesions. Unfortunately, the activity of these infections at the time of the study could not be defined using positron emission tomography with fluorodeoxyglucose integrated with computed tomography (FDG‐PET/CT); however, all but 2 patients could be considered having active infection from the results of serology followed over time (AE1 and AE6; Table 2).^25^ Serology results at the time of performing the whole‐blood test were as follows: 9/12 AE subjects were tested with HCF‐EIA and EgP‐EIA, and among them 7/9 had positive results in both tests; a positive AgB‐EITB result was found in 3/3 patients; positive results to Em2plus and to Em18 were found in 7/9 and 4/5 AE patients, respectively. The results of the whole‐blood IL‐4 test in comparison with serology are shown in Table 2. Whole‐blood test indeterminate results were obtained in 2/12 patients with AE; no obvious explanations were found for such results in the clinical records of the patients. These patients were excluded from further evaluations. Among the 10 AE mitogen‐responding patients, the IL‐4 levels ranged from 0 pg/mL to 0.07 pg/mL [median 0, interquartile range (IQR): 0‐0.03 pg/mL] (Figure 2). When we compared the robustness of the results obtained in this study with those previously published by Petrone et al^12^ by comparing the IL‐4 levels in response to the SEB‐positive control, no significant differences were found (*P* = .91) (Figure 2). Based on the IL‐4 cut‐off in response to the AgB‐enriched fraction of 0.39 pg/mL,^12^ none of the 10 AE patients scored positive to the whole‐blood test. Therefore, as shown in Table 3, the previously found overall sensitivity for CE diagnosis was 71%^12^; here, we add further preliminary results suggesting that the specificity of the test (not assessed in Petrone et al^12^) may be excellent (100% in our cohort) to rule out AE.

**Table 1:** Demographic and clinical characteristics of the enrolled subjects

N (%)	12 (100.0)
Median age in years (IQR)	66 (46‐70)
Female gender N (%)	6 (50.0)
Origin N (%)	
Western Europe	12 (100.0)
Eastern Europe	‐
Asia	‐
North America	‐
Previous medical treatment N (%)	11 (91.7)
Previous surgical resection N (%)	4 (33.3)
Lesions N (%)	
Liver	9 (75.0)
Liver plus other localizations	3 (25.0)

Abbreviations: IQR, interquartile range; N, number.

**Table 2:** Detailed description of the patients enrolled

PT	Age	Gender	Lesion localization based on US and/or CT imaging	Albendazole therapy duration (years)	Surgery (years before enrolment)	Whole‐blood IL‐4 test		Serology					
						Indeterminate results	AgB results	HCF ELISA	EGP ELISA	AgB‐EITB	EM2PLUS ELISA	EM11 ELISA	EM18 ELISA
AE1	68	Female	Liver, myocardium, pericardium	2	2	No	Negative	Positive	Positive	NA	Positive	NA	Positive
AE2	68	Female	Liver	2	2	No	Negative	Negative	Negative	NA	Negative	NA	Negative
AE3	72	Male	Liver	4	‐	No	Negative	NA	NA	NA	Positive	Positive	NA
AE4	52	Male	Liver	1	‐	No	Negative	Positive	Positive	NA	Positive	NA	Positive
AE5	66	Male	Liver	2	5	No	Negative	Negative	Negative	NA	Negative	NA	NA
AE6	33	Male	Liver	8	7	Yes	Negative	NA	NA	NA	Positive	NA	NA
AE7	30	Female	Liver	0	‐	Yes	Negative	Positive	Positive	Positive	NA	Positive	NA
AE8	66	Female	Liver, abdominal wall	10^a^	‐	No	Negative	Positive	Positive	NA	NA	NA	Positive
AE9	76	Male	Liver, diaphragm, vena cava into right atrium	2^b^	‐	No	Negative	Positive	Positive	Positive	NA	Negative	Positive
AE10	82	Female	Liver	4	‐	No	Negative	NA	NA	Positive	Positive	NA	NA
AE11	65	Female	Liver	1	‐	No	Negative	Positive	Positive	NA	Positive	NA	NA
AE12	81	Male	Liver	1	‐	No	Negative	Positive	Positive	NA	Positive	NA	NA

Note

Patient AE5 received albendazole for 2 years, and the treatment was discontinued 3 years before performing whole‐blood IL‐4 test; all other patients were receiving albendazole at the time of testing.

Abbreviations: AE, alveolar echinococcosis; AgB, antigen B; CT, computed tomography; HCF, hydatid cyst fluid; IL, Interleukin; NA, not available; PT, patient; US, ultrasound.

a Days.

b Months.

**Table 3:** Comparison of the whole‐blood test sensitivity for CE or AE diagnosis

Diagnosis	Petrone et al^12^ N = 57 (mitogen responding)	Present study N = 10 (mitogen responding)
	Whole‐blood test positive	Whole‐blood test positive
CE	30/42 (71.4%)	NA
Healthy donors	1/15 (6.7%)	NA
AE	NA	0/10 (0%)

Abbreviations: AE, alveolar echinococcosis; CE, cystic echinococcosis; N, number; NA, not available.

## 4 DISCUSSION

The cross‐reactivity between AE and CE is an important limitation of the serological tests used to complement the diagnosis of these two infections, with serious consequences in clinical practice.^4^ In our work, 78% of patients with AE had positive results in one or more tests based on *E granulosus* antigens; in contrast, our preliminary results interestingly showed that none of AE patients in our cohort had a positive whole‐blood IL‐4 test. These preliminary data suggest a high specificity of the whole‐blood test based on an AgB‐enriched fraction, which may be able to allow ruling out AE in case of a positive result. Strikingly, although AgB from *E granulosus* and *E multilocularis* shows a high amino acid homology,^26^ the whole‐blood test, based on a T‐cell response, may allow the identification of *E granulosus*‐specific epitope regions not recognized by IgG antibodies. These deserve future identification to develop recombinant antigens and therefore a standardized whole‐blood assay.

This preliminary study has several limitations. The patients number was limited, although it must be taken into account that AE is a rare condition and only 104 new cases were reported throughout Europe in 2016.^27^

Almost all AE patients were receiving treatment at the time of testing, and standardized assessment of infection activity, for example through FDG‐PET/CT, could not be performed. The inactivity of the lesions indeed may have affected the whole‐blood negative results in AE patients. However, the hallmark of *E multilocularis* infection is the secretion of regulatory cytokines, as IL‐10 and TGF‐β, which are also observed in patients with AE, especially in those with advanced and severe disease,^28^ suggesting that the absence of IL‐4 production may even actually indicate lesion activity. Further studies on treatment‐naïve patients and other patients groups with standardized assessment of infection activity would be required to ascertain the impact of the pharmacological treatment and lesion activity on the whole‐blood assay results. Lack of protein integrity was not responsible of this absence of IL‐4 response in our cohort as indicated by SDS‐PAGE analysis (Figure 1). Finally, immunosuppression from HIV or immunosuppressive therapy was excluded based on inclusion criteria and all patients were deemed immunocompetent based on their response to the mitogen.

Several data regarding the serology of AE are missing. This is due to the study having been conducted in parallel with the routine clinical practices, which included serological testing off‐site and did not included systematic serial serological testing. The unavailability of sera for research purposes also did not allow us to test antibody responses to AgB‐enriched fraction in sera from AE patients, as a parallel assessment to our primary aim.

Furthermore, it was not possible in our setting to directly compare the results of the test from CE‐infected and AE‐infected patients, as CE is extremely rare in Switzerland. The findings could be only compared with previous results on patients with CE.^12^ Moreover, differences observed in the CE and AE cohorts do not take into account the variability of AgB‐enriched fraction preparations used. Indeed, AgB subunits are highly polymorphic and protein composition may vary from one purification to another. However, our results support that the AgB‐enriched fraction used in these assays is a good antigen preparation (data not shown) also because the Western blot analysis of the AgB major bands appears as multiples of 8kDa (i.e. 8, 16, 24, 32 kDa) (Figure 1) and because we know that AgB represents about 90% of the immunodominant antigens purified from hydatid cyst fluid.^29^ Further evaluations using a standardized source of AgB are needed to confirm our findings. Nevertheless, our preliminary results encourage further studies to be performed, especially in CE‐AE co‐endemic areas.

In conclusion, our preliminary data show that the IL‐4 whole‐blood test is a promising assay to rule out AE in case of test positivity, in the context of a differential diagnosis with CE. This finding, combined with its potential usefulness in discriminating active from inactive CE stages 11, 12 and the possibility to standardize the test, underscores this assay's potential to further improve the diagnosis of CE.

## DISCLOSURES

None.

## AUTHOR CONTRIBUTION

LP performed the IL‐4 ELISA, analysed and interpreted data, and wrote the manuscript; WCA, MH enrolled patients with AE; WCA, MH and MF collected clinical data; MAGM provided AgB‐enriched fraction and participated in the interpretation of data; AT participated in the interpretation of data; FT, WCA and MH participated in the interpretation of data and contributed to the writing of the manuscript; DG designed the study, coordinated and supervised the project, contributed to the interpretation of the results, contributed to the writing of the manuscript. All authors discussed the results and approved the final version of the manuscript.

## Data Availability

The data that support the findings of this study are available from the corresponding author upon reasonable request.
